# Cognitive evaluation in unaccompanied refugee children: a systematic review

**DOI:** 10.1590/1984-0462/2023/41/2022079

**Published:** 2023-05-15

**Authors:** Dienifer Katrine Chierici, Amer Cavalheiro Hamdan

**Affiliations:** a Universidade Federal do Paraná, Curitiba, PR, Brazil.

**Keywords:** Child, Refugee, Psychological tests, Cognition, Systematic review, Criança, Refugiado, Testes psicológicos, Cognição, Revisão sistemática

## Abstract

**Objective:**

This study aims to identify what existing literature has shown about possible cognitive alterations in unaccompanied refugee children.

**Data sources:**

The search was performed in the Web of Science, PsycInfo, Scopus, and PubMed databases, including articles published in any year and in any language. The research was submitted to the Prospero protocol (ID: CRD42021257858), and the quality of the included articles was evaluated using the Mixed Methods Appraisal Tool.

**Data synthesis:**

Memory and attention are the main topics identified, largely because they are related to symptoms of post-traumatic stress disorder. However, low specificity was observed in the conduction of cognitive assessments, leading to relevant inconsistencies in the collected data.

**Conclusions:**

The use of psychological assessment instruments that are either poorly adapted or not adapted at all to the populations studied casts doubt on the validity of the data produced so far.

## RESUMO

**Objetivo:**

Identificar o que a literatura tem apresentado a respeito de possíveis alterações cognitivas em crianças refugiadas desacompanhadas.

**Fontes de dados:**

Foi realizada uma busca nas bases de dados Web of Science, PsycInfo, Scopus e PubMed, que incluiu artigos indexados produzidos em qualquer período e em qualquer idioma. A pesquisa foi submetida ao protocolo do *International Prospective Register of Systematic Reviews* — Prospero (ID: CRD42021257858), e a qualidade dos artigos selecionados foi avaliada por meio do *Mixed Methods Appraisal Tool*.

**Síntese dos dados:**

Memória e atenção foram os principais tópicos identificados, em grande parte por sua relação com sintomas de transtorno do estresse pós-traumático. Contudo, foi observada baixa especificidade na condução dos testes cognitivos, levando a importantes inconsistências entre os dados coletados.

**Conclusões:**

O uso de instrumentos de avaliação psicológica mal adaptados ou inadaptados para as populações estudadas coloca em dúvida a validade dos dados produzidos.

## INTRODUCTION

By the end of 2020, 82.4 million people had been forced to relocate from their country of origin due to persecution, conflict, violence, human rights violations, or events seriously disturbing the public order.^1^ Of these, 35 million (42%) are under the age of 18 years and 1 million were already born refugees. Venezuela currently represents 86.2% of the total number of legal refugees in Brazil, and in 2020, most Venezuelan refugee applicants were under 15 years old.^2^

Unaccompanied children, specifically, refer to those who are separated from both parents and other relatives and are not being cared for by any adult who, by law or custom, is responsible for doing so.^3^ It is important to emphasize that the concept of adolescence included in this review is based on the definition of “child,” adopted by the Convention on the Rights of the Child, as a person under the age of 18 years.^4^

During the process of forced migration, individuals are exposed to multiple vulnerabilities and potentially traumatic situations. Often, these are people who have lost all their belongings, do not speak the local language, and have no access to food, housing, work, education, or health services.^5^ In addition, they are exposed to various situations of xenophobia, racism, exploitation, as well as physical, sexual, and psychological violence. Among unaccompanied refugee children, limited social support networks and the precarious legal situation in which they find themselves are also a threat to adequate child development.^6^

Given this exposure to many risk factors, refugee children are more prone to developing psychological disorders. In a previous review on the topic, Kien et al.^7^ identified among refugee children rates of anxiety between 8.7 and 31.6%, depression between 10.3 and 32.8%, and post-traumatic stress disorder (PTSD) between 19 and 52.7%, while the prevalence of PTSD in the general population of children ranges between 2 and 9%. In addition, the authors highlight significant levels of learning disorders, addictions, hyperactivity, aggressive and challenging behavior, social withdrawal, and clinically relevant somatic symptoms.

In the same vein, the literature review by Kaplan et al.^8^ suggests that experiencing traumatic events in childhood can cause cognitive, emotional, and behavioral changes that affect learning, academic performance, and performance on intelligence tests. Furthermore, traumatic experiences in childhood are associated with impairments in memory, attention, executive functions, and abstract reasoning.^9^ The authors speculate that symptomatic reactions to traumatic events may be associated with impairment in cognitive functioning. As an example, they cite PTSD symptoms (insomnia, hypervigilance, stress, etc.) as directly or indirectly responsible for changes in cognitive and school performance.

Kaplan et al.^8^ observed that family functioning is also related to cognitive development. Thus, children who are separated from their families may be exposed to yet another risk factor for the development of deficits in cognitive performance. Despite the above, Kaplan et al.^8^ did not identify research that directly investigates the correlation between cognitive changes and children coming from refuge situations. It is important to highlight that the absence of studies investigating this population may make the emotional and cognitive particularities of children in situations of refuge invisible.

The assessment of cognitive functions in refugee children is especially complex, because the instruments used need to go through a careful process of cross-cultural adaptation. A proper adaptation would include the processes of translation, evaluation of the translation by experts, evaluation of the translation by the target audience, reverse translation, and a pilot study.^10^ In a systematic review, Gadeberg et al.^11^ identified low levels of evidence and validation in tools used for the psychological assessment of refugee children. The authors highlight the urgency of developing validated tools for such a population, stating that the focus has been primarily on adults. They also pointed out that the use of non-validated tools undermines clinical assessments and the outcome of scientific studies, resulting in the pathologization of healthy individuals or the neglect of those who need further follow-up and treatment. For all those reasons, this systematic review aimed to analyze what the literature has evidenced so far regarding possible cognitive changes in unaccompanied refugee children. To the best of our knowledge, there are no previous studies dealing with this subject.

## METHOD

This is a systematic review study, conducted according to the *Preferred Reporting Items for Systematic Reviews and Meta-Analyses* (PRISMA) guidelines.^12^ The study protocol was submitted to the PROSPERO (*International Prospective Register of Systematic Reviews*) platform under the registration number CRD42021257858.

The inclusion criteria adopted were empirical studies, without language or publication date restrictions, available online or in university library systems. Descriptive studies, systematic or literature reviews, letters to the editor, chapters or full books, opinion texts, and corrections were excluded. Articles that assessed cognition in accompanied children and articles that did not assess cognition were also excluded.

Initially, it was proposed to exclude articles that assessed cognition in children who did not have legal refugee status. However, due to the complexity of the topic and the ambiguous nature of the refugee status definition itself, it was decided to include in the sample two studies with children coming from war situations, but who were not properly recognized as refugees.

The review was conducted in the electronic databases Web of Science, PsycInfo, Scopus, and PubMed on May 29, 2021. The following descriptors were used, with their formula adapted to the requirements of each database: (“refugee” AND “unaccompanied” OR “orphan”) AND (“cognition*” OR “impairment” OR “deficit” OR “memory” OR “attention” OR “perception” OR “language” OR “executive function”). The filter “document type (article)” was selected in all databases that have it. A second query of the same databases was performed on July 9, 2021, replacing the descriptor “refugee” with terms used for the undocumented refugee population, “asylum seeker” and “internally displaced”.

The selection process of the studies occurred in two stages. In the first stage, the articles found were read and selected based on their titles and abstracts, as well as on their adequacy to the previously established inclusion and exclusion criteria. In the second stage, the selected articles were read in their entirety, and their suitability to the study objectives was reevaluated based on the article’s main objectives, study design, and sample characteristics. Finally, the publications selected for review were assessed for quality criteria by applying the Mixed Methods Appraisal Tool (MMAT checklist).^13^ The methodological quality of each study was rated according to the following parameters: low, between 0 and 25% adequacy to the applied assessment tool, regular between 26 and 50%, good between 51 and 85%, and great at values higher than 85%.

## RESULTS

**Figure 1.**
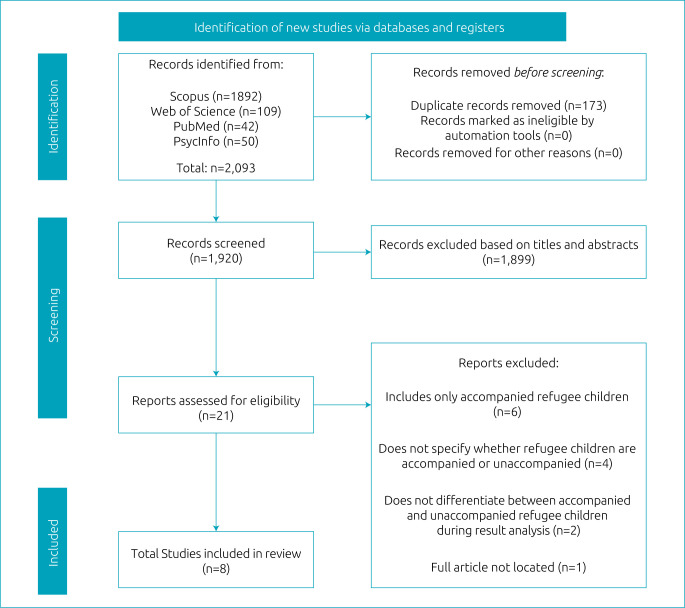
PRISMA flowchart for the inclusion of articles.

Figure 1 shows the article selection process. In total, in the first stage, 21 articles (out of 2,093 results) were selected for full reading. We chose to include one study that does not distinguish between accompanied and unaccompanied children in its data analysis, in which the sample was composed of 80% unaccompanied refugee children. At the end of the process, eight articles were selected. The complementary search, conducted on July 9, 2021, in the same databases, did not result in the inclusion of additional studies to the sample.

Table 1 shows the main characteristics of the included studies. It is important to note that only 50% of the articles had the main objective of evaluating cognitive functions, with the others having obtained data on cognition through secondary results during the application of tests. Faced with this scenario, we chose to make a cut, presenting only the data directly related to the objective of this review. In addition, part of the results referring to cognition were obtained through the use of tests that were not originally developed for this purpose, and it is possible to observe great incompatibility between some of the tests used and the cognitive function evaluated. However, it is noteworthy that all studies sought to provide justifications for the methodological choices adopted and bring some level of discussion about the cognitive function evaluated.

**Table 1.** Characterization of the included articles regarding objectives, study design, samples, countries of origin, and main findings.

Author, year	Study design	Sample	Countries of origin	Conclusions
Childs, 2021^14^	Case-control	8 M/5 F Age: 16–23 years *Mean age:* 18±2 years	Sudan (23%), Afghanistan (15%), Ethiopia (15%), Eritrea (15%), Albania (8%), Cameroon (8%), Somalia (8%), Vietnam (8%)	Separated youth had significantly lower IQ scores. No differences in verbal memory were observed compared to the control group.
Wolff et al., 1995^15^	Case-control	39 M/35 F Age: 4–7 years *Mean age:* 5.7 years	Eritrea	Orphans showed not only higher cognitive performance than the accompanied refugee children but also greater behavioral symptoms. No children showed language deficits.
Huemer, 2012^16^	Case-control	35 M/6 F Age: 15–18 years *Mean age:* 17±1 years	Somalia, Algeria, Nigeria, Gambia, Ghana, Niger, Guinea-Bissau, Mali, Eritrea, and Kenya	Significant positive correlation between attention problems and symptoms of stress, anger, and anxiety.
Pfeiffer, 2019^17^	Cross-sectional	334, 90.7% M *Mean age:* 16±2 years	31 countries, including Afghanistan (42%), Syria (19.1%), Eritrea (6.2%), Somalia (6.2%), Gambia (4.1%), Iraq (2.9%), Iran (2.6%) Guinea (2.1%), Morocco (1.4%), Albania (1.2%), Ethiopia (1.2%), and Nigeria (1.2%)	Investigating post-traumatic stress disorder, topological overlaps between sleep disturbances and concentration problems (sustained attention) were indicated.
Spinhoven, 2006^18^	Longitudinal	920 (73% M) Age: 12–18 years *Mean age:* 17±2 years	48 countries, predominantly Angola (43%), Sierra Leone (10%), and China (8%)	Memory inconsistencies do not differ from other population groups.
Wolff and Fesseha, 1999^19^	Case-control	20 M/20 F Age: 9–12 years *Mean age:* 11 years	Eritrea	Orphans scored more than unaccompanied refugee children on most cognitive tests. There was no difference in performance on the Raven’s Matrices or the Token Test.
Huemer, 2016^20^	Case-control	35M/6F Age: 15–18 years *Mean age:* 17±1 years	Somalia, Algeria, Nigeria, Gambia, Ghana, Niger, Guinea-Bissau, Mali, Eritrea, and Kenya	Highly emotionally charged verbal content, but significantly lower word count than the control group.
Longobardi, 2017^21^	Cross-sectional	18 M/1 F Age: 16–17 years	Egypt (26.3%), Albania (26.3%), Senegal (15.8%), Bangladesh (10.5%), Gambia (10.5%), Morocco (5.3%), Mali (5.3%)	Attention results within the reference range (normal).

M: male; F: female.

The sample consists of papers published between 1995 and 2021 in the English language, with a prevalence of studies conducted in England (n=3) and Austria (n=2). All papers were conducted and published in European countries. Males accounted for 74.2% of the sample of these articles, which reflects an important research bias. According to the UN High Commissioner for Refugees,^1^ the percentage of children in forced displacement in 2020 was equivalent for both genders. As for study designs, there was a predominance of case-control studies (62.5%), followed by longitudinal (25%) and cross-sectional studies (12.5%).

Regarding the origin of the studied population, only one of the articles does not inform precisely the composition of its sample, reporting only that 48 countries were included and that 48% of the sample came from Angola, 10% from Sierra Leone, and 8% from China. Furthermore, five articles have a sample exclusively from the African continent. The remaining two articles have a mixed sample composed of children from Africa and the Middle East and correspond to the most recently published articles. Eritrea is the country of origin more represented in the studies (62.5%), followed by Gambia (50%) and Somalia (50%), Albania (37.5%), Mali (37.5%), and Nigeria (37.5%).

The age of the participants was in the range of 4–23 years (mean of 14.5 years, with the omission of one study where these data were not informed). Standard deviation is present in only 6.5% of the studies, with a mean value of 1.4 years. One study included unaccompanied persons aged up to 23 years, but who obtained refugee status before the age of 18 years. Another study included participants aged up to 21 years, justifying that in their country (Germany) these persons still live in foster homes and are treated in children’s hospitals. The remaining articles included individuals within the traditional age range of up to 18 years.

No agreement or standardization was identified in the psychological assessment instruments used by different groups of researchers. The tests used in more than one article (Table 2) were the Youth Self-Report (YSR) (n=2), The Raven Progressive Matrices (n=2), Token Test (n=2), and Expressive One-Word Picture Vocabulary Test (n=2). However, such instruments identified in more than one study refer to research by the same author.

**Table 2.** Characterization of articles regarding the instruments used and the cognitive functions assessed.

Author, year	Instruments	Cognitive functions
Childs, 2021^14^	*Gudjonsson Suggestibility Scale* (GSS); *Wechsler Abbreviated Scale of Intelligence* (WASI)	Memory, intelligence
Wolff et al., 1995^15^	*The Leiter International Intelligence Scale; The Raven Progressive Matrices; Token Test (Short Term); Grooved PegBoard Test for Children; Expressive One-Word Picture Vocabulary Test*	Intelligence, perception, language, memory, and attention
Huemer, 2012^16^	*Weinberger Adjustment Inventory* (WAI); *Youth Self-Report* (YSR)	Attention
Pfeiffer, 2019^17^	*The Child and Adolescent Trauma Screen* (CATS)	Attention
Spinhoven, 2006^18^	*Hopkins Symptom Checklist* (HSCL); *Reactions of Adolescents to Traumatic Stress Questionnaire* (RATS); *Stressful Life Events Questionnaire* (SLE)	Memory
Wolff and Fesseha, 1999^19^	*The Kaufman Assessment Battery for Children* (KABC); *The Raven Progressive Matrices; Token Test (Short Term)*; *Expressive One-Word Picture Vocabulary Test*	Intelligence, memory, executive functions, perception, and language
Huemer, 2016^20^	*Mini-International Neuropsychiatric Interview for children and adolescents (MINI Kid); Youth Self-Report* (YSR); *UCLA Child/Adolescent PTSD Reaction Index; Facts About You; Stress-Inducing Speech Task* (SIST)	Language, memory
Huemer, 2016^20^	*Mini-International Neuropsychiatric Interview for children and adolescents* (MINI Kid); *Youth Self-Report* (YSR); *UCLA Child/Adolescent PTSD Reaction Index; Facts About You; Stress-Inducing Speech Task* (SIST)	Language, memory
Longobardi, 2017^21^	*Strengths and Difficulties Questionnaire* (SDQ); *Trauma Symptom Checklist for Children* (TSCC)	Attention

Memory is the cognitive function most frequently represented in the analyzed sample, being present in 62.5% of the studies, followed by attention in 50% of the studies. Both were predominantly assessed with instruments usually aimed at identifying trauma and general maladaptation. All the cognitive functions predicted in this review were evaluated in at least one study, and in addition, there was the identification of evaluations measuring the intelligence factor, which was not initially predicted.

Regarding the methodological quality, assessed using the MMAT checklist,^13^ only two studies (25%) achieved optimal scores, namely, Childs et al.^14^ and Wolff et al.^15^ Four studies (50%) achieved a good score, namely, Huemer et al.,^16^ Pfeiffer et al.,^17^ Spinhoven et al.,^18^ and Wolff and Fesseha,^19^ and two other studies (25%) scored a methodological quality considered regular, namely, Huemer et al.^20^ and Longobardi et al.^21^ Major problems were the use of inappropriate measurement techniques (75% of the studies) and response rates below what is considered acceptable, factors that compromise the generalization of obtained results. Table 3 summarizes the quality evaluation based on the MMAT.

**Table 3.** Assessment of the methodological quality of studies based on the Mixed Methods Appraisal Tool (MMAT checklist).

Study designs	Authors	Are the participants representative of the target population?	Are there complete outcome data?	Are measurements appropriate regarding both the outcome and intervention?	During the study period, is the intervention administered (or exposure occurred) as intended?	Results (%)
Quantitative nonrandomized	Childs, 2021^14^	Yes	Yes	Yes	Yes	100
	Wolff et al., 1995^15^	Yes	Yes	Yes	Yes	100
	Wolff and Fesseha, 1999^19^	Yes	Yes	No	Yes	75
	Huemer, 2016^20^	Yes	No	No	Yes	50
		**Is the sample representative of the target population?**	**Is the sampling strategy relevant to address the research question?**	**Are the measurements appropriate?**	**Is the statistical analysis appropriate to answer the research question?**	**Results**
Quantitative descriptive	Huemer, 2012^16^	Yes	Yes	No	Yes	75
	Pfeiffer, 2019^17^	Yes	Yes	No	Yes	75
	Spinhoven, 2006^18^	Yes	Yes	Yes	No	75
	Longobardi, 2017^21^	Yes	Yes	No	No	50

## DISCUSSION

This review sought to analyze what are the possible cognitive alterations identified in the population of unaccompanied refugee children. The data showed that memory and attention are the main focus of the reviewed studies, an interest largely related to the high incidence of PTSD in the refugee population. However, the forms of assessment were often inadequate, and the results proved inconsistent in most cases.

Regarding memory, Childs et al.,^14^ Wolff et. al.,^15^ Wolff and Fesseha,^19^ and Spinhoven et al.^18^ found no significant changes. However, it is interesting to note that Spinhoven et al.^18^ identified a negative association between the level of trauma and the number of inconsistencies in memory, suggesting the existence of a psychological adjustment mechanism responsible for such compensation. Also addressing the relationship between trauma level and memory, Huemer et al.^20^ suggested problems in the integration of autobiographical memories related to emotional factors, corroborating with the study of Mueller et al.^22^

Regarding attention, Longobardi et al.^21^ found performance within the expected range. Wolff et al.^15^ found no significant differences between the orphaned and unaccompanied refugee population. On the contrary, Huemer et al.^16^ identified a positive correlation between levels of stress, anger, and anxiety with attention problems. In the same vein, Pfeiffer et al.^17^ suggested a strong association between sleep disturbances and concentration problems (sustained attention), pointing out that this is one of the three main symptoms of PTSD. The results of Huemer et al.^16^ and Pfeiffer et al.^17^ corroborate the proposition by Kaplan et al.^8^ that symptomatic reactions to traumatic events may be related to cognitive malfunction, highlighting the importance of further investigations.

As for language, Wolff et al.^15^ and Wolff and Fesseha^19^ observed no significant differences between orphan and unaccompanied refugee child samples. On the contrary, Huemer et al.^20^ identified slower and more emotionally charged verbal production, especially among those with higher levels of trauma. Childs et al.,^14^ 2021, Wolff et al.,^15^ and Wolff and Fesseha^19^ assessed intelligence and observed decreased intellectual capacity compared to control subjects. Previous research has documented lower levels of intellectual functioning and verbal ability among children with traumatic life experiences.^23-25^ However, barriers imposed by foreign language, literacy, and cultural variables present during cognitive assessment need to be considered when interpreting these results.^8^

Finally, only one study assessed executive functions indirectly as part of their battery for intelligence assessment.^19^ The results showed significantly lower performance, even with the use of nonverbal tests. The same was true for perception.^15,19^ There are few studies concerning perception and executive functions in the studied population, combined with an important temporal gap, since the last publication on the subject dates from 1999.

From the above, it is evident that the evaluation of cognitive functions in the population of unaccompanied refugee children is a topic that has been little explored. The large number of inconsistencies between the identified results suggests the need for studies with a higher degree of specificity regarding the cognitive domains studied, in order to identify more precisely their possible alterations.

In addition, the problem of using culturally inappropriate instruments for the psychological assessment process of refugee populations is highlighted. The application of the MMAT checklist^13^ verified that most of the reviewed articles did not address this issue. This was largely due to lack of translation, lack of standardized measures, validation, or standards for ethnically similar samples. As highlighted earlier, adequate measures would include translation, expert translation assessment, target audience translation assessment, reverse translation, and pilot study. Gadeberg et al.^11^ had already identified the low level of evidence and validation in tools targeting this audience. As the authors pointed out, the use of poorly adapted instruments that are not sensitive to cultural variants may jeopardize the conclusions and generalization of the results.

In general, the focus of studies has been on comparisons between unaccompanied refugee children and children native to the host country. No articles were identified that investigated the differences between those who obtained refuge with or without the presence of their caregivers, so the influence of this variable on cognition remains unknown. Kaplan et al.^8^ suggested that family functioning has an important influence on cognitive development. Thus, it is speculated that this variable interferes with the child’s school and community adaptation in their new country, as well as with the early identification of changes in cognitive performance.

Moreover, the data on the country of origin of the samples provide important information about how scientific research on migratory phenomena has been carried out. The refugee crisis in the Middle East intensified in the year 2015, and therefore, it is interesting to note that only more recent studies (2019 and 2021) began to include Eastern subjects in their samples. Furthermore, it is relevant to note that no studies have included subjects originating from Latin American and Caribbean countries, despite these localities hosting one of the largest refugee crises today¹. Such a latency period between the occurrence of the phenomena and their respective scientific investigation may represent a limitation to accessing data such as short-term disturbances in cognitive functioning, in addition to making the available data insensitive to cultural differences between populations.

The main methodological limitation of this article is the inclusion of studies that adopted indirect or de-standardized cognitive assessment methodologies, an occurrence that limits the reliability and generalization of the data obtained. However, such inclusion reflects the reality of a precarious scientific scenario for the psychological assessment of refugee populations, giving voice to the important discussion on this issue. Furthermore, the methodological choice to restrict the study population to unaccompanied children may have limited access to important data on cognitive assessment of the child refugee population. Given that parents and guardians play a large role in identifying cognitive changes and seeking professional follow-up, accompanied refugee children may be more likely to participate in scientific studies and receive appropriate cognitive assessments.

The assessment of cognitive functions in unaccompanied refugee children is a little explored theoretical and practical field, with a limited number of published articles, and is often investigated indirectly. Memory and attention functions are the most frequently represented, largely because they are related to symptoms of PTSD. This, in turn, is widely investigated in the studied population, but the articles rarely pay attention to the cognitive implications caused by the disorder.

This review may help future research that aims to study cognitive functions in the population of refugee children. It is recommended that studies with a higher degree of specificity regarding the evaluated cognitive function be carried out, with the appropriate cross-cultural adaptation of instruments for the psychological evaluation of children. We verified a low number of studies exploring issues related to perception and executive functions, combined with an important temporal gap. This finding opens an invitation for new researchers to investigate the subject.

Finally, neurocognitive psychodiagnosis has been gaining an increasing space within the health area, given the importance of early identification of dysfunctions, the development of intervention plans, and cognitive training for a better prognosis of the patient. Besides the cultural variables, when referring to the refugee population, we are dealing with a public in a situation of multiple vulnerabilities. This implies the need for caution and responsibility in the use of psychological testing tools, in order to enable accurate, reasoned, and ethical interventions.
